# Linear and Nonlinear Guided Wave Imaging of Impact Damage in CFRP Using a Probabilistic Approach

**DOI:** 10.3390/ma9110901

**Published:** 2016-11-07

**Authors:** Jan Hettler, Morteza Tabatabaeipour, Steven Delrue, Koen Van Den Abeele

**Affiliations:** Group Wave Propagation and Signal Processing, KU Leuven Kulak, Etienne Sabbelaan 53, 8500 Kortrijk, Belgium; morteza.tabatabaeipour@kuleuven.be (M.T.); steven.delrue@kuleuven.be (S.D.); koen.vandenabeele@kuleuven.be (K.V.D.A.)

**Keywords:** ultrasonic, guided waves, RAPID, baseline-free, SHM

## Abstract

The amount and variety of composite structures that need to be inspected for the presence of impact damage has grown significantly in the last few decades. In this paper, an application of a probabilistic ultrasonic guided wave imaging technique for impact damage detection in carbon fiber-reinforced polymers (CFRP) is presented. On the one hand, a linear, baseline-dependent, technique utilizing the well-known correlation-based RAPID method and an array of piezoelectric transducers is applied to detect impact-induced damage in plate-like composite structures. Furthermore, a baseline-independent nonlinear extension of the standard RAPID method is proposed, and its performance is demonstrated both numerically and experimentally. Compared to the conventional RAPID, the baseline-free version suffers from a somewhat lower imaging quality. However, this drawback is compensated by the fact that no damage-free (intact) baseline is necessary for successful imaging of damage.

## 1. Introduction

Conventional nondestructive testing (NDT) methods, such as visual inspection, ultrasonic C-scan, thermography or shearography, are well established and frequently used in the field of quality control and defect inspection. Unfortunately, as one can imagine from an example, such as an ultrasonic C-scan of a composite aircraft wing, these methods are quite bulky and time consuming. Unlike point-by-point inspection techniques, guided wave (GW) techniques used for material characterization and evaluation are driven by their ability to inspect large areas using only a limited amount of ultrasonic transducers. Therefore, the use of GW-based NDT could significantly decrease the inspection time and costs compared to conventional NDT methods. Moreover, the transducers within the transmitting-receiving network can be kept in place during the operational lifetime of the component, because they are small enough not to influence the mechanical performance of the component. Thanks to the improvement in technology, the durability of such systems becomes more and more recognized. Therefore, GW-based methods represent a perfect choice for structural health monitoring (SHM) and defect imaging.

Lamb waves are one type of guided waves usually associated with elastic wave propagation in thin plates. First described in 1917 by British mathematician Horace Lamb in [1], they have drawn attention ever since. Worlton [2,3] was perhaps the first author who recognized the potential of Lamb waves for NDT. Shortly after, Grigsby [4] described and highlighted the most important properties of Lamb waves with respect to the inspection of thin plates. However, the modern era of Lamb wave research and practical applications dates back to the influential book written by Viktorov in 1967 [5]. Since then, Lamb waves found their way into many different fields. In NDT only, they have been used for the inspection of plates [6,7,8], pipelines [9,10], rails [11], steel wire ropes [12,13,14] and other complex structures, such as aircraft wings and fuselage panels [15,16,17,18]. In our contribution, we will focus solely on the application of the Lamb waves for the defect imaging in the plate-like structures.

Although several attempts to visualize defects using the *filtered back-projection* tomographic algorithm were done [19,20,21,22,23,24,25], the most popular alternative methods are the sum-and-delay methods and the probability-based algorithms. Both approaches rely on information obtained from a sparse array or network of ultrasonic transducers that are in most cases permanently attached to the inspected sample. The sparse array usually consists of a variable number of small, but efficient piezoelectric transducers (PZT) [26]. In the present work, our focus is aimed at the second approach, represented here by the RAPID algorithm.

RAPID, first introduced in 2005 by Gao et al. [27], employs a probability-based algorithm and database of signal difference coefficients (SDCs) to localize and image defects in the plate-like sample. First, the SDC calculation is used to quantify the difference between a signal acquired in the intact state and a signal acquired after damage was introduced to the sample [17,28]. Next, the SDC of each transmitter-receiver (T-R) pair is projected on the corresponding path between the transmitting and receiving elements of the sparse array, and all contributions are summed to provide a map of the damage indicator (damage index) [29,30]. This way, the damage is visualized and localized within the area defined by the sparse array network. One of the most important disadvantages of RAPID, due to its baseline-dependent nature, is the inherent problem of optimal baseline selection, because changes in environmental conditions during a measurement can result in false indications. Several authors proposed different measures to improve the robustness of RAPID to varying environmental conditions, for example by considering a shape factor optimization [31] or a GW mode selection [32]. However, the issues with environmental interference still persist. Hence, in this contribution, we propose a nonlinear baseline-free modification of RAPID that addresses these issues.

This paper is ordered as follows: Section 2 is dedicated to the description of the conventional RAPID algorithm and its nonlinear baseline-free modification. Next, we present the results of numerical simulations that were carried out to assess the performance of this baseline-free modification. The fourth section describes the experimental imaging results of the conventional and the baseline-free RAPID on an orthotropic CFRP plate. The contribution is summarized and the conclusions are made in the last section.

## 2. Guided Wave Imaging Using RAPID

**Figure 1:**
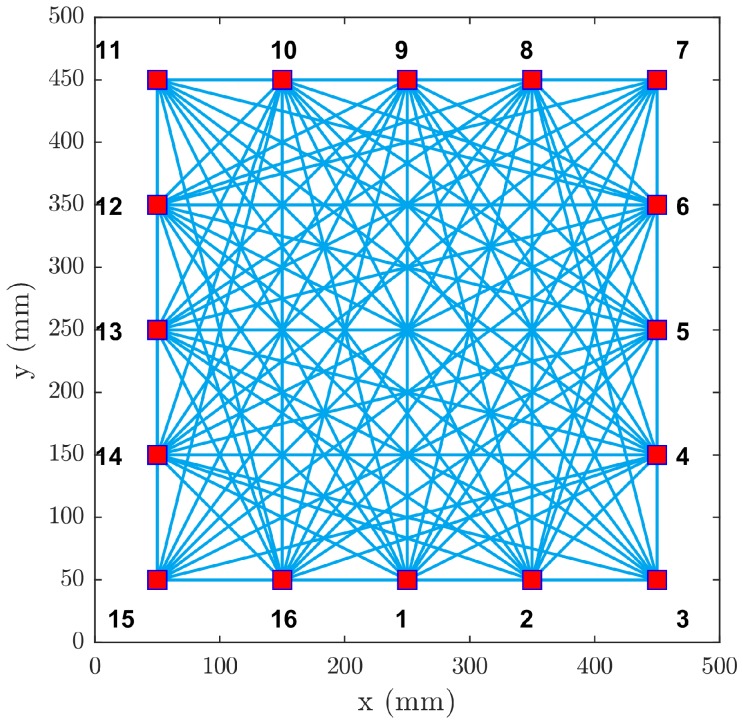
Array coverage with 16 PZTs.

Conventional RAPID, as described by Gao et al. [27], utilizes data from an ultrasonic sparse array consisting of $n_{e}$ permanently-attached PZT transducers. The direct line-of-sight coverage of such an array is visualized in Figure 1. In the conventional RAPID, signals between each transmitter-receiver pair are acquired in two different states: baseline (intact, without damage) and damaged (after the damage has been introduced) [28]. Let the signal transmitted from the sparse array element *i* to element *j* be denoted $B_{ij}$ and $D_{ij}$ for the baseline and damage state, respectively. If the sparse array consists of $n_{e}$ elements, then the total number of acquired signals is $n_{e}\left(n_{e}-1\right)$. The total number of signals can be reduced down to $\frac{n_{e}}{2}\left(n_{e}-1\right)$, if the reciprocity of the system ($B_{ij}=B_{ji}$) is assumed [17,30].

**Figure 2:**
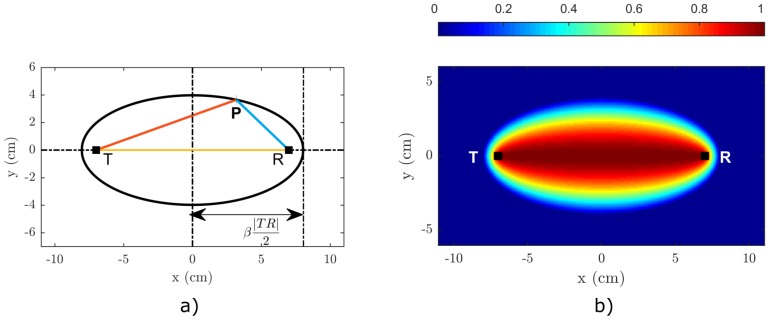
Geometrical interpretation of the (**a**) $R_{ij}\left(x,y\right)$ function and (**b**) $s_{ij}\left(x,y\right)$ function.

Fundamentally, the RAPID algorithm can be broken down into two major parts: the SDC calculation and the imaging. The SDC values represent a measure of the dissimilarity between each two signals obtained in two different states, i.e., between $B_{ij}$ and $D_{ij}$. Even though being the second step in the algorithm, the imaging part will be described first, since it is general and common for both the baseline-dependent (linear) and baseline-free (nonlinear) version of the algorithm. Let us first thus assume that the $SDC_{ij}$ coefficients are known for all T-R pairs. We start by dividing the inspected area of the sample into a rectangular equidistant mesh. Next, we define a priori probability distribution $s_{ij}$ (see Figure 2b) for each T-R pair and every point [x,y] of the mesh as follows:

(1) $$s_{ij}\left(x,y\right)=\left\{\begin{array}{cc} \frac{\beta -R_{ij}\left(x,y\right)}{\beta -1}, & \mathrm{if}\;\beta >R_{ij}\left(x,y\right)\\ 0, & \mathrm{if}\;\beta \leq R_{ij}\left(x,y\right) \end{array}\right.,$$

where *β* stands for a free-to-choose shape factor that defines the area influenced by one T-R pair [33]. $R_{ij}\left(x,y\right)$ is a geometrical function defined as:

(2) $$R_{ij}\left(x,y\right)=\frac{\sqrt{{\left(x_{i}-x\right)}^{2}+{\left(y_{i}-y\right)}^{2}}+\sqrt{{\left(x_{j}-x\right)}^{2}+{\left(y_{j}-y\right)}^{2}}}{\sqrt{{\left(x_{j}-x_{i}\right)}^{2}+{\left(y_{j}-y_{i}\right)}^{2}}}.$$

which is representing the ratio of the distances |TP|+|PR| and the |TR|. As a result, the numerator $\beta -R_{ij}\left(x,y\right)$ from (1) actually describes an ellipse with focal points located in *T*, *R* and a major axis length $\beta \frac{\left|TR\right|}{2}$, as illustrated in Figure 2. This ellipse forms the borderline of the region influenced by a particular T-R pair. If a selected point *P* lies within the area defined by the borderline, its $s_{ij}$ decreases with the distance from the straight line that connects points *T* and *R*. In other words, the further the point *P* from the direct path between the transmitter and the receiver, the lower its $s_{ij}$ value will be.

The $s_{ij}$ is evaluated for all T-R pairs and at all points of the rectangular grid. Once combined with the individual $SDC_{ij}$ values, the final damage index heat map is calculated as:

(3) $$P\left(x,y\right)=\sum_{i=1}^{n_{e}}\sum_{j=1,i\neq j}^{n_{e}}SDC_{ij}s_{ij}\left(x,y\right),$$

where contributions from all T-R pairs are summed up at a particular grid point with the $SDC_{ij}$ values serving as geometrical weights. A cluster of points with a high damage index $P_{ij}$ then indicates the potential location of a flaw in the sample.

### 2.1. Linear RAPID

In the case of the conventional implementation of RAPID, the $SDC_{ij}$ is calculated using the standard correlation coefficient defined as:

(4) $$\rho_{ij}=\frac{\mathrm{Cov}(B_{ij},D_{ij})}{\sigma \left(B_{ij}\right)\sigma \left(D_{ij}\right)},$$

where $\sigma (B_{ij})$ and $\sigma (D_{ij})$ are the signal variances and:

(5) $$\mathrm{Cov}\left(B_{ij},D_{ij}\right)=\sum_{k=1}^{n_{s}}\left(B_{ij}\left[k\right]-\mu_{ij}^{B}\right)\left(D_{ij}\left[k\right]-\mu_{ij}^{D}\right),\;i,j=1,2,\cdots ,n_{e},i\neq j$$

defines the covariance of the baseline and the damaged signals. Values $\mu_{ij}$ denote the mean value of the corresponding signal between transmitter *i* and receiver *j*, and the index [k] simply indicates that all signals are discretely sampled at a sampling rate of $f_{s}=\frac{1}{\Delta_{t}}$ (Δt is the sampling interval). The $SDC_{ij}$ value that quantifies the difference in signals can then be calculated using $\rho_{ij}$ as:

(6) $$SDC_{ij}=1-\rho_{ij},\;i,j=1,...,n_{e},\;i\neq j.$$

The lower the correlation between the baseline and damaged signals, the higher the SDC coefficient will be and the more profound the dissimilarity is between the two signals.

The basic idea behind the use of SDC values for imaging purposes can be illustrated with the following example. Imagine that a damage has been introduced to a sample after the acquisition of baseline signals. The presence of the damage triggers changes in the wave propagation through the sample, and if the damage lies on or close to a direct path between a selected T-R pair of the network, the wave propagation will be altered significantly, resulting in a rise of the corresponding $SDC_{ij}$ (correlation decreases). However, if the damage is sufficiently far from the sensor pair, its influence on the wave propagation will be negligible, and the corresponding $SDC_{ij}$ value is low. Thus, by combining the input from all T-R pairs, the damage index $P_{ij}$ map can be constructed, and the damage can be visualized.

### 2.2. Nonlinear RAPID

The main advantages and disadvantages of the conventional RAPID methodology, outlined in the previous section, were already presented in the Introduction. It is clear that the weakest spot of this technique is the baseline signal selection process. Changes in the environmental conditions, at which the $B_{ij}$ and $D_{ij}$ were measured, can result in severe deterioration of the imaging quality. Hence, in this section, we propose a method that transforms the conventional RAPID into a baseline-free method by introducing a SDC parameter based on the scaling subtraction method (SSM) [34,35]. This will eliminate the requirement for an intact baseline signal, which should improve the real-world applicability of RAPID.

First, some major assumptions have to be presented before the method itself is described. It is assumed that:

- the defect behaves nonlinearly with increasing excitation amplitude, meaning that it responds differently to a low amplitude excitation than to a high amplitude excitation due to the existence of a mesoscopically nonlinear stress-strain behavior at the defect location.
- the nonlinearity of the equipment and transducers is low.

These assumptions have to be made, otherwise the new localization concept would make no sense. If the nonlinearity of the transmitter and receiver is higher than the nonlinearity of the defect, the imaging would simply fail. Since the nonlinearity of the transducer response depends on the excitation amplitude, this has to be kept sufficiently low not to provoke a distortion of the transmitted signal. This acceptable level can be found experimentally by direct monitoring of the output waveform distortion as a function of input amplitude.

The details of the baseline-free RAPID method are as follows. First, a low amplitude excitation signal is applied to the sparse array transducers, and the corresponding response signals $B_{ij}$ are obtained. The response signals $B_{ij}$ will act as reference (defect-free) signals. Next, a high amplitude excitation is applied, and the response signals $D_{ij}$ are acquired under the same measurement conditions. Both sets of measurements can be obtained at the same time (a matter of seconds difference). Hence, the influence of the environmental conditions, such as temperature variation or mechanical loading, is negligible. The only difference is that the excitation amplitude has been up-scaled by a scaling factor:

(7) $$k_{s}=\frac{A_{D}}{A_{B}},$$

where $A_{B}$, $A_{D}$ are the amplitudes of the excitation signals for low and high amplitude, respectively. The new, nonlinearity-based, SDC coefficient is given by the mean square difference between the high amplitude response signals and the up-scaled low amplitude response:

(8) $$SDC_{ij}=\frac{1}{n}\sum_{m=1}^{n}{\left(k_{s}B_{ij}\left(t_{m}\right)-D_{ij}\left(t_{m}\right)\right)}^{2}.$$

If the inspected system is purely linear, the response signals scale up perfectly and are simply equal to $k_{s}\;\cdot \;B_{ij}$. Hence, the value of the SDC will be equal to zero in this case. The imaging will result in a blurred unfocused image without any defect indication. However, if a nonlinear defect is present in the interrogated sample, the SDC value attains a non-zero value for some of the T-R paths.

Based on this principle, the SDC in (3) can be simply replaced by Equation (8), and the new RAPID method basically becomes baseline-independent, because it does not need an input from a state measured before the damage took place.

### 2.3. Correction for Direct Path Propagation

The original RAPID algorithm brings about a second problem. For the imaging to be successful, the SDC value for each T-R pair should be calculated only from the part of the recorded signal that corresponds to the direct propagation path (extended by parameter *β*) between the transducers. This can be easily done in isotropic materials, but it is considerably more difficult to fulfil this restriction in orthotropic samples.

In order to restrict the useful signal and include only the direct propagation, a simple manipulation can be suggested for the received signal. Assuming an anisotropic plate, let us define the distance between the transmitter and receiver by *x* and the angle of propagation with respect to the coordinate system of the anisotropic plate by *θ*. Furthermore, we assume that the entire recorded signal contains $n_{s}$ samples. To assure that only the direct propagation is taken into account, a reduced amount of samples $n_{r}$ can be considered. Here, $n_{r}$ ($n_{r}<n_{s}$) can be easily calculated from an estimate of the TOF between transmitter *i* and receiver *j* as follows:

(9) $$n_{r}=\left\lfloor TOF_{ij}f_{s}\right\rfloor +\frac{n_{c}}{2}\frac{f_{s}}{f},$$

where $f_{s}$ is the sampling frequency and $n_{c}$ is the number of cycles in the excitation waveform at a frequency *f*. $TOF_{ij}$ is the time-of-flight defined as:

(10) $$TOF_{ij}=\frac{x}{c_{ph}\left(\theta \right)},$$

where $c_{ph}\left(\theta \right)$ is the phase velocity of the selected GW mode in the direction given by the angle of propagation *θ* in the anisotropic plate. As can be seen from (9), a number of cycles $n_{c}$ that is equal to the arrival time of the center of the wave packet is taken into account. The phase velocity can be determined from numerical dispersion data, provided most of the elastic constants of the sample are known. This approach is used to ensure a proper imaging strategy in anisotropic materials.

### 2.4. Damage Index Image Processing

The resulting damage index P(x,y) heat map can be improved by thresholding the SDC values prior to being used in the imaging step. In order to do this, the SDC values are first rescaled to a closed interval [0,1]. Next, the values below a specified threshold are set to zero, and the remaining ones are left intact. Clearly, the number of contributing T-R pairs that form the image will drop, and only the most influenced ones remain. In a more advanced manner, the value of the threshold $t_{SDC}$ can be determined empirically or linked to the mean value and standard deviation of the initial SDC values. The general description of this thresholding operation is:

(11) $$SDC_{ij}=\left\{\begin{array}{cc} 0 & \mathrm{if}\;SDC_{ij}<t_{SDC}\;i,j=1,2,...,n_{e},i\neq j\\ SDC_{ij} & \mathrm{if}\;SDC_{ij}\geq t_{SDC}\;i,j=1,2,...,n_{e},i\neq j \end{array}\right..$$

Finally, in order to facilitate an easier visual perception, the resulting image can be simply thresholded at a fixed level $t_{P}$:

(12) $$P\left(x,y\right)=\left\{\begin{array}{cc} 1 & \mathrm{if}\;P\left(x,y\right)<t_{P}\\ 0 & \mathrm{if}\;P\left(x,y\right)\geq t_{P} \end{array}\right.,$$

thereby creating a binary image that immediately pinpoints the areas with the highest damage index $P_{ij}$. If a fixed threshold is applied to the result, the size and location of the suspected defect(s) can be easily estimated.

## 3. Numerical Simulations

Numerical simulations were carried out in order to verify the proposed baseline-free RAPID methodology. The simulations were performed using COMSOL Multiphysics^©^ (COMSOL AB, Stockholm, Sweden).

### 3.1. Model

**Figure 3:**
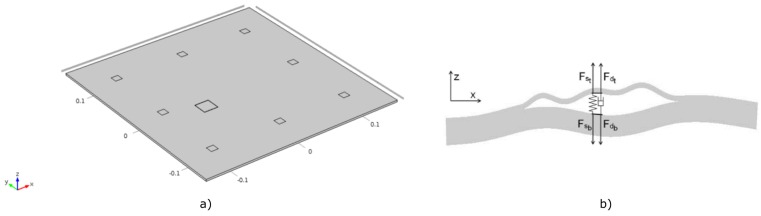
(**a**) Numerically-simulated anisotropic plate; (**b**) spring-damper model of a delamination.

The test sample was simulated as a simple bulk square CFRP T300/924C plate with orthotropic symmetry and dimensions of 288 mm × 296 mm × 2.7 mm (see Figure 3a). The density of the material is $\rho =1548\;\mathrm{kg}\;m^{-3}$, and its elastic properties are summarized in Table 1. The simulation model includes damping that was implemented using a Rayleigh damping model, which is a standard included in COMSOL Multiphysics^©^. In our case, the mass damping parameter $\alpha_{dM}$ of the model was set to zero, and the stiffness damping parameter:

(13) $$\beta_{dK}=\frac{1}{2\pi fQ}$$

was defined based on the quality factor *Q*. The quality factor was chosen to be constant (Q=20) for all simulations.

**Figure 4:**
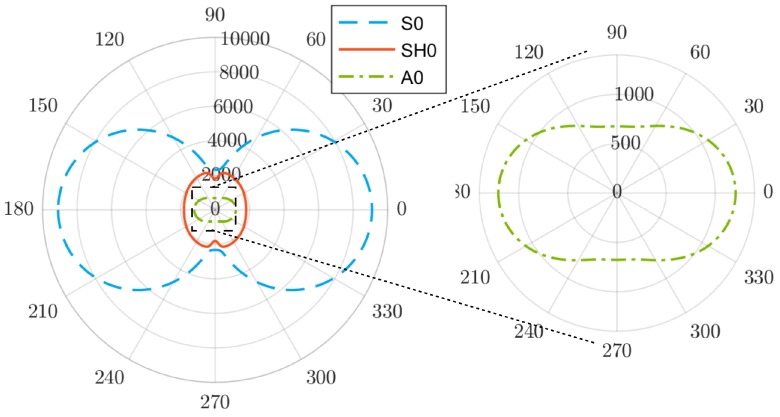
Angular profile of the phase velocity $c_{ph}$ at 50 kHz for the simulated plate (T300/924C).

Figure 4 shows the dependency of the phase velocity on the angle of propagation for the fundamental GW modes at a fixed frequency of 50 kHz (calculated using the Legendre polynomial approach [36]). These data will be used to carry out the direct propagation correction.

**Figure 5:**
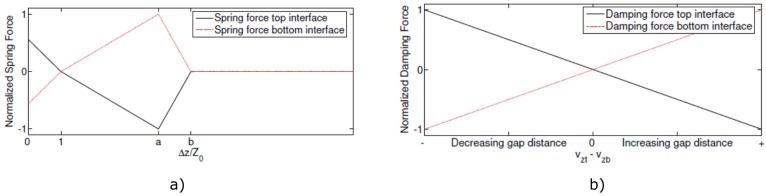
(**a**) Spring forces $F_{st}$ and $F_{sb}$ and (**b**) damping forces $F_{dt}$ and $F_{db}$ at the simulated delamination as a function of the gap distance ΔZ [38].

For the simulations, the sample contained either one or two (depending on the configuration) 20 mm × 20 mm nonlinear delaminations located at 1/4th of the plate’s thickness (see Figure 3a). Each defect was simulated as a clapping system (kissing bond) based on the nonlinear stress-strain model developed by Delrue and Van Den Abeele [37]. The graphical representation of this model is illustrated in Figure 3b. The nonlinear dynamic behavior of the delamination is controlled by a set of virtual spring and damper forces at both sides of the delamination, as illustrated in Figure 3b. These forces can be expressed as functions of the gap distance ΔZ. Above a certain separation threshold, the two sides are completely separated (stress free surfaces); below the threshold, particular formulations of the van der Waals forces are implemented. When the surfaces are close to each other, they will be attracted to each other. However, when they tend to be too close, the attraction force will turn into a repulsive force, trying to separate the surfaces again. The piecewise behavior of these additionally introduced elastic contact forces for a kissing bond is illustrated in Figure 5.

Apart from the elastic contact force, damping forces were also implemented, which are acting against the velocity of separation. These forces make sure that the surfaces of the delamination are not opening too abruptly, avoiding a destruction of the material. At the same time, they assure that the surfaces are not closing to fast so that the two surfaces cannot overlap. These forces are shown in Figure 3b. The nonlinear viscoelastic behavior at the delamination level mimics the clapping behavior of a delamination. Depending on the displacement amplitude of the wave passing by the defect, this nonlinearity will be activated or not, creating a distortion in the wave propagation, which can be measured in the received signal after appropriate signal processing.

The implementation of the nonlinear spring-damper forces is performed by introducing dynamic boundary conditions in COMSOL Multiphysics^©^, at those nodes that correspond to the delamination surface. At these positions, the nodes are split in pairs, and the following analytical formula’s are implemented for the spring and damper forces [37]:

(14) $$F_{s_{t}}=-F_{s_{b}}=\left\{\begin{array}{cc} k_{1}\left(Z_{0}-\Delta z\right) & \mathrm{if}\;\Delta z<Z_{0}\\ k_{2}\left(Z_{0}-\Delta z\right) & \mathrm{if}\;Z_{0}\leq \Delta z<aZ_{0}\\ k_{3}\left(bZ_{0}-\Delta z\right) & \mathrm{if}\;aZ_{0}\leq \Delta z<bZ_{0}\\ 0 & \mathrm{if}\;bZ_{0}\leq \Delta z \end{array}\right.,$$

(15) $$F_{d_{t}}=-F_{d_{b}}=\left\{\begin{array}{cc} -\gamma (v_{z_{t}}-v_{z_{b}}) & \mathrm{if}\;\leq \Delta z<bZ_{0}\\ 0 & \mathrm{if}\;\Delta z\geq bZ_{0} \end{array}\right.,$$

where $Z_{0}$ is a small characteristic distance between the faces of the delamination, *a*, *b* are free parameters defining the separation conditions (1<a<b), *γ* is a damping coefficient and $v_{z_{t}}$ and $v_{z_{b}}$ stand for the normal velocities of the top and bottom interfaces. $k_{1}$, $k_{2}$ and $k_{3}$ are the virtual spring constants that are connected by:

(16) $$k_{3}=\frac{1-a}{b-a}k_{2}.$$

Details on the parameter values for the nonlinear delamination model that was used in the numerical simulation are summarized in Table 2.

### 3.2. Results

In order to test the viability of the proposed baseline-free modification, four numerical evaluate cases were defined. In the test cases, we test the performance of the imaging algorithm using a variable number and locations of delaminations and different forms of excitation. The test cases are summarized in Table 3.

The imaging was carried out using a sparse network of eight virtual transducers placed in a rectangular array. The locations of the transducers are depicted in Figure 3a. The excitation waveform consisted of a 20-cycle ($n_{c}=20$) Hanning windowed sine burst at 50 kHz. The excitation was implemented as a prescribed normal displacement at the area of the transmitting virtual element. The received signal was sampled with a sampling frequency of $f_{s}=10\;\mathrm{MHz}$. The adaptive signal length correction was applied using the dispersion data from Figure 4.

**Figure 6:**
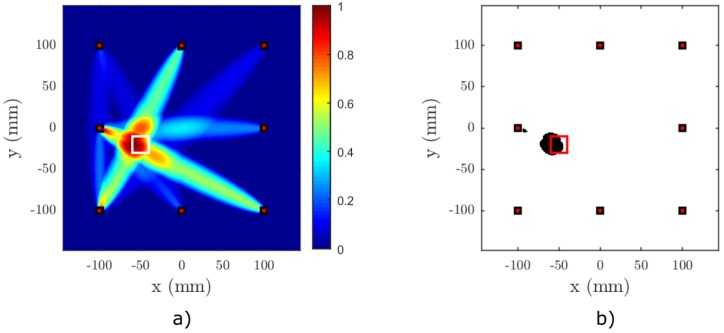
Result of Test Case 1 for nonlinear RAPID imaging on a simulated anisotropic plate, (**a**) without binarization and (**b**) with binary thresholding. The center of the delamination is located at [−50,−20]. The binarization threshold was set to $t_{P}=0.8$, the shape factor to β=1.015 and the SDC threshold to $t_{SDC}=0.25$. The actual location of the delamination is marked with a white square in (a) and a red square in (b).

**Figure 7:**
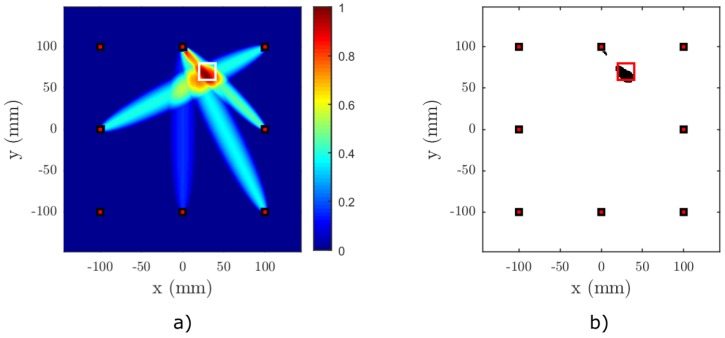
Result of Test Case 2 for nonlinear RAPID imaging on a simulated anisotropic plate, (**a**) without binarization and (**b**) with binary thresholding. The center of the delamination is located at [30,−70]. The binarization threshold was set to $t_{P}=0.8$, the shape factor to β=1.015 and the SDC threshold to $t_{SDC}=0.25$. The actual location of the delamination is marked with a white square in (a) and a red square in (b).

**Figure 8:**
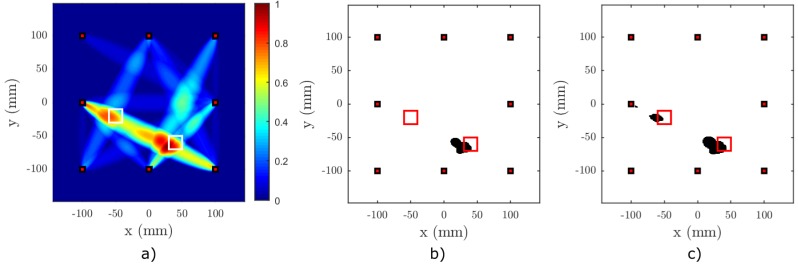
Result of Test Case 3 for nonlinear RAPID imaging on a simulated anisotropic plate, (**a**) without binarization, (**b**) with binary thresholding ($t_{P}=0.8$) and (**c**) with lowered binary threshold ($t_{P}=0.75$). The centers of the delaminations are located at [−50,−20] and [40,−60]. The shape factor is set to β=1.015 and the SDC threshold to $t_{SDC}=0.25$. The actual location of the delamination is marked with white squares in (a) and a red square in (b,c).

**Figure 9:**
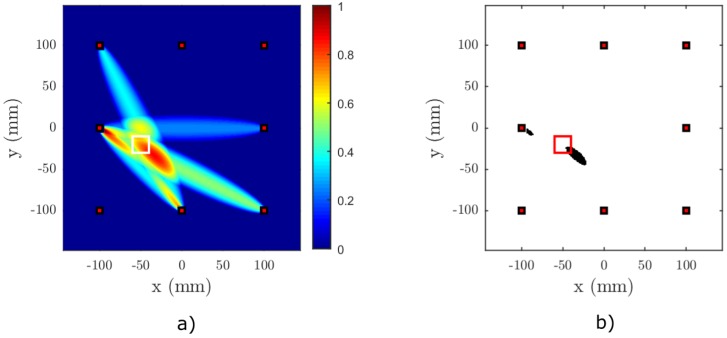
Result of Test Case 4 for nonlinear RAPID imaging on a simulated anisotropic plate, (**a**) without binarization and (**b**) with binary thresholding. The excitation waveform was a three-cycle sine burst, at 50 kHz with the Hanning window. The center of the delamination is located at [−50,−20]. The binarization threshold was set to $t_{P}=0.8$, the shape factor to β=1.015 and the SDC threshold to $t_{SDC}=0.25$. The actual location of the delamination is marked with a white square in (a) and a red square in (b).

Figure 6, Figure 7, Figure 8 and Figure 9 show the results of the baseline-free RAPID detection for a CFRP plate containing a single or two nonlinear delaminations with the properties described in the previous subsection. Except for Test Case 3, all delaminations were successfully detected, as can be clearly seen from the binarized images. In Test Case 3, due to the mutual position of the sparse array elements and the defects, it was necessary to lower the threshold by 5% in order to successfully detect both delaminations (see Figure 8c). However, it can be easily observed from Figure 8a that both defect zones are clearly highlighted.

The fourth and last test case demonstrates the effect of signal duration (number of sine cycles) on the imaging performance. The test case is similar to the first test case with one exception. The number of cycles in the excitation signal was decreased from $n_{c}=20$ to $n_{c}=3$. The effect of this change can be observed in Figure 9. It is obvious from a comparison between Figure 6 and Figure 9 that the imaging output is different for different values of $n_{c}$. A higher number of cycles clearly improves the performance of the baseline-free imaging algorithm.

The main reason for the inferior imaging performance at low $n_{c}$ is the fact that a shorter signal is inherently more broadband, and the more broadband the signal is, the higher the dispersion will be. As a natural consequence, we obtain a less precise estimation of the TOF and a poorer adaptive signal length correction. On the other hand, a larger $n_{c}$ instigates unavoidable problems with reflections coming back from the edges of the plate. Therefore, an optimal $n_{c}$ that avoids reflections and provides narrowband excitation has to be found for every application, based on the sample geometry and the sample’s dispersion characteristics.

Another reason may be linked to the behavior of the nonlinear delamination and the related SDC value. At low $n_{c}$, only a very small portion of the signal is distorted due to the nonlinear delamination, because the faces of the delamination undergo just three cycles instead of 20. Therefore, the nonlinear effects influence the result only slightly at $n_{c}=3$, whereas they have more time to build up for $n_{c}=20$. According to Delrue and Van Den Abeele [37], the combination of SSM and the nonlinear delamination model performs very well for larger $n_{c}\approx 40$. The rigorous study of the mode behavior for the small $n_{c}$ has not been published yet.

## 4. Experimental Results

The above discussed linear and nonlinear versions of RAPID were further experimentally verified on an orthotropic CFRP plate.

### 4.1. Test Sample

**Figure 10:**
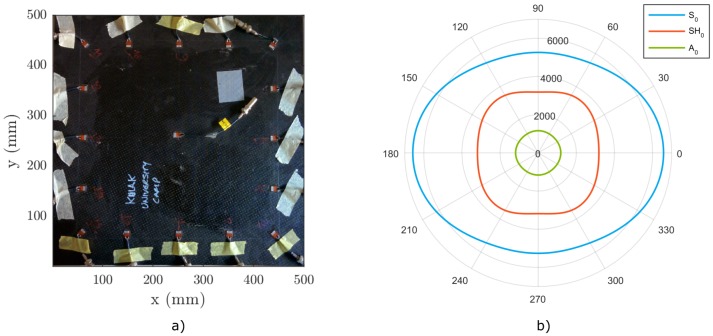
(**a**) CFRP plate for experimental testing; (**b**) angular dependence of the phase velocity at 50 kHz for the CFRP plate.

The test sample to be investigated is a 400 mm × 400 mm CFRP plate consisting of 11 separate layers/plies (see Figure 10a). The stacking sequence of the laminate, as well as the ply type are given in Table A1. The laminated composite can be concisely described using the standard stacking sequence notation as ${\left[{\left(0,45\right)}_{2},90,-45,0,-45,90,0\right]}_{T}$ [39]. The elastic properties of the plies are collected in Table A2. The total thickness of the plate is approximately $h_{tot}=4.3\;\mathrm{mm}$. Due to the (non-symmetric) stacking sequence, the global (homogenized) in-plane elastic properties of the plate cannot be assumed simply quasi-isotropic. Isotropy can only be assumed for the lowest antisymmetric mode $A_{0}$, as can be seen in Figure 10b.

The angular profile of $c_{ph}$ for the $A_{0}$ mode is nearly circular. The phase velocities of the other modes, such as $S_{0}$ and $SH_{0}$, are strongly dependent on the direction of propagation. This is a fact that has to be kept in mind during the damage location or beam-forming imaging of laminated plates with non-symmetric layups.

### 4.2. Experimental Setup

For the experimental validation of RAPID, an experimental setup for GW imaging was developed, consisting of a single channel arbitrary waveform generator (AWG) NI PXI-5412 (100 MHz, 14-bit, single channel, National Instruments Corporation, Austin, TX, USA) that was routed to a multiplexing unit via an AR150A100B amplifier (Amplifier Research, Souderton, PA, USA). The signal from the switch board was connected to the individual PZTs of the sparse array network using a 50 Ω shielded coaxial cable. The input from the amplifier is connected to the transmitting element, while the other elements are connected to the receiving digitizers: NI PXI-5122 (100 MHz, 14-bit, two channels, National Instruments Corporation, Austin, TX, USA). All transmitting and receiving cards were hosted in a single chassis NI PXI-1022 (National Instruments Corporation, Austin, TX, USA). The transmitting element is disconnected from the receiving stage during the excitation to avoid leakage and crosstalk of this signal to other channels. The PZT transducers used as sparse array elements were DuraAct^©^ P-876 flexible PZTs (PI Ceramic GmbH, Lederhose, Germany), in either rectangular or circular shape, covered in a protective polymeric layer. The entire measurement was controlled using a virtual instrument (VI) program written in LabVIEW^©^.

### 4.3. Linear Conventional RAPID

**Figure 11:**
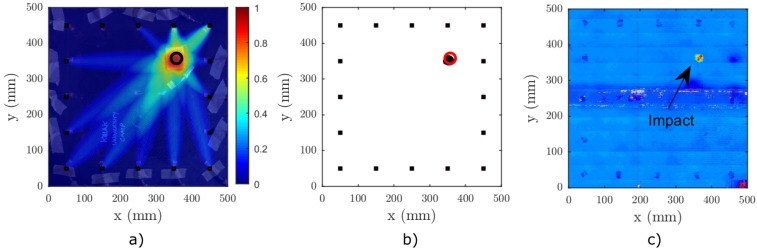
Baseline-dependent RAPID imaging on a CFRP plate, excitation waveform: five-cycle sine burst f=50 kHz, β=1.015 and $t_{SDC}=0.5$ (**a**) damage index P(x,y), (**b**) thresholded binary RAPID image using a threshold level at $t_{P}=0.9\max\left\{P\left(x,y\right)\right\}$ (**c**) ultrasonic C-scan of the plate.

In order to illustrate the conventional baseline dependent RAPID, a test was conducted on a CFRP plate. It was equipped with 16 rectangular DuraAct^©^ transducers (PI Ceramic GmbH, Lederhose, Germany) that were coupled using Hysol^©^ adhesive (Henkel AG, Düsseldorf, Germany). The baseline signal was recorded at standard room temperature in laboratory conditions using the experimental setup described in Section 4.2. It was subsequently damaged by a 21 J impact in the upper right corner (see Figure 11c for the result of a traditional C-scan analysis after impact).

Before and after the impact, with a difference of more than a month, the sample was excited using a five-cycle sine burst at 50 kHz with a Hanning window. According to Figure 10b, only the $A_{0}$ and $S_{0}$ modes are excited at this frequency range. Moreover, using the experimental dispersion analysis, it was found that the $A_{0}$ mode is the most dominant mode at this excitation frequency. The received signals were sampled at 10 MHz, and $n_{s}$ = 16,384 samples were acquired. The input amplitude to the amplifier was 0.1 V. The “current” set of signals $D_{ij}$ was acquired using the same test stand at ambient room temperature without any precise temperature adjustments.

For the subsequent analysis, the length of the signal between every T-R pair was restricted to include only the direct propagation of the $A_{0}$ mode [32]. This was easily done based on the angular dispersion data and the phase and group velocities from Figure 10b. Hence, knowing the propagation distance, direction and velocity, the signal can be properly time windowed.

The resulting damage index image is depicted in Figure 11a. The impact zone is well indicated in the binarized image (Figure 11b).

### 4.4. Nonlinear Baseline-Free RAPID

The experimental setup for the nonlinearity-based baseline-free RAPID validation was the same as described in Section 4.2. The lower input amplitude to the amplifier was set to $A_{lin}=0.01\;V$, and the SSM scaling coefficient was $k_{s}=10$. The actual voltage input to the PZT was approximately 8 V and 80 V for the lower and higher amplitude excitation, respectively. The excitation frequency was f=50 kHz, and the waveform was a three-cycle Hanning windowed tone burst. Such a low cycle count was selected due to the position of the defect and due to the shape of the sparse array. A longer duration of excitation would result in a mixing of directly propagating signals and signals that are reflected from the edges of the sample. Hence, the short excitation is unavoidable in this case. The signals were sampled with a sampling frequency $f_{s}=10\;\mathrm{MHz}$, and each signal was averaged 512 times to get a good SNR for the low amplitude excitation.

**Figure 12:**
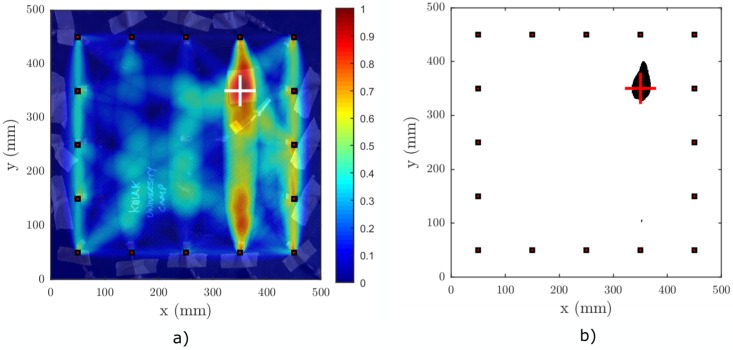
Result of nonlinear RAPID imaging on a CFRP plate, (**a**) without binarization and (**b**) with binary thresholding. The excitation waveform was a three-cycle sine burst at 50 kHz with a Hanning window. Threshold for binarization was set to $t_{P}=0.8$. The actual location of the delamination is marked with a white square in (a) and a red square in (b).

Figure 12 shows the result of the nonlinear RAPID imaging of the impact damage, applying the following imaging parameters: $t_{SDC}=0.15$, β=1.015, $t_{P}=0.8$. The defect was satisfactorily highlighted in the damage index map and subsequently segmented using the fixed threshold binarization.

## 5. Discussion and Conclusions

Based on the linear and nonlinear measurements and subsequent analysis, we come to the following conclusions: First, the conventional linear RAPID, employing correlation-based SDC coefficients, proved to be a considerably reliable imaging technique when used in the laboratory conditions. The predicted location of the damage corresponds very well with the ultrasonic C-scan data depicted in Figure 11c. On the other hand, the size of the defect is somewhat overestimated. However, it is a very satisfactory result especially considering the extended period between which the baseline and damaged signals were taken.

Second, the nonlinear baseline-free RAPID was able to detect the same defect using SSM-based SDC coefficients. Important to note here is that the coefficient and the baseline signals were acquired after the impact damage was introduced in the plate and that no reference to an intact state is required. Figure 12 suggests that most of the nonlinear effects are generated when waves propagate through the damaged region in the *y*-direction. This can be attributed to the distribution of the partial damage caused by the impact along the dominant material symmetry axes (0°, 90°). However, the shape of the damage distribution inferred by the nonlinear RAPID method does not entirely conform with the result from the ultrasonic C-scan. Therefore, another technique with higher spatial resolution, for instance uCT, should be used to confirm whether the micro-cracking is indeed present in a wider area than predicted by the C-scan.

Compared to the conventional RAPID, the baseline-free version suffers from lower imaging quality. However, this drawback is compensated by the fact that no damage-free (intact) baseline is necessary for successful imaging of damage. This promising result can be further investigated and developed in view of achieving more robust and more environment interference-resistant SHM methods in the future.

## Figures and Tables

**Table 1:** Material properties of the T300/924C composite.

Young’s Modulus [GPa]	Shear Modulus [GPa]	Poisson’s Ratio
$E_{xx}=127.1$	$G_{xy}=5.0$	$\nu_{xy}=0.320$
$E_{yy}=8.34$	$G_{yz}=2.7$	$\nu_{yz}=0.461$
$E_{zz}=8.85$	$G_{zx}=4.8$	$\nu_{zx}=0.461$

**Table 2:** Model parameters of the nonlinear delamination.

Parameter Name	Value	Unit
$Z_{0}$	0.1	mm
*a*	4	
*b*	5	
$k_{1}$	$5\times 10^{13}$	${\mathrm{Nm}}^{-3}$
$k_{2}$	$5\times 10^{12}$	${\mathrm{Nm}}^{-3}$
*γ*	$10^{7}$	${\mathrm{Nsm}}^{-3}$

**Table 3:** Specifications of the numerical test cases.

Test Case	Delaminations	Locations	$A_{B}$ [nm]	$k_{s}$ [nm]	$n_{c}$
1	1	[−50,−20]	10	100	20
2	1	[30,70]	10	100	20
3	2	[−50,−20], [40,−60]	10	100	20
4	1	[−50,−20]	10	100	3

## Abbreviations

The following abbreviations are used in this manuscript:

ACU	Air-coupled ultrasound
AWG	Arbitrary waveform generator
CFRP	Carbon fiber-reinforced polymer
GW	Guided wave
NDT	Nondestructive testing
PZT	Piezoelectric transducer
RAPID	Reconstruction algorithm for probabilistic inspection of damage
SDC	Signal difference coefficient
SHM	Structural health monitoring
SSM	Scaling subtraction method
TOF	Time-of-flight

## Appendix A

**Table A1:** Stacking sequence of a CFRP plate. Angle *φ* defines the in-plane rotation of the ply with respect to the global coordinate system (first Euler angle). The total thickness of the plate is 4.3 mm.

Ply #	Type	*φ* [°]	$h_{i}$ [mm]	*ρ* [$\mathrm{kg}\;m^{-3}$]
1	5 Harness	0	0.480	1770
2	NCFBiaxial	45	0.480	1790
3	Uni-weave	0	0.480	1790
4	NCF Biaxial	45	0.480	1790
5	NCF Biaxial	0	0.480	1790
6	NCF Biaxial	90	0.480	1790
7	NCF Biaxial	−45	0.480	1790
8	Uni-weave	0	0.480	1790
9	NCF Biaxial	−45	0.480	1790
10	5 Harness	90	0.480	1770
11	Glass Ply	0	0.1270	2500

**Table A2:** Engineering elastic properties in the principal directions for the CFRP layered plate.

Lamina	$E_{1}$	$E_{2}$	$E_{3}$	$G_{12}$	$G_{23}$	$G_{13}$	$\nu_{12}$	$\nu_{13}$	$\nu_{23}$
	[GPa]			[GPa]			[-]		
5 Harness	65.0	65.0	8.8	3.6	3.6	3.6	0.041	0.041	0.041
NCF Biaxial	81.0	81.0	8.8	4.1	4.1	4.1	0.033	0.033	0.033
Uni-weave	152.0	8.8	8.8	4.1	4.1	4.1	0.018	0.018	0.310
Glass ply	70.0	70.0	70.0	28.7	28.7	28.7	0.220	0.220	0.220
